# Intolerance of Uncertainty and Emotional Processing in Adolescence: Separating Between-Person Stability and Within-Person Change

**DOI:** 10.1007/s10802-022-01020-1

**Published:** 2023-01-27

**Authors:** Marco Lauriola, Sara Iannattone, Gioia Bottesi

**Affiliations:** 1 Department of Social and Developmental Psychology, Sapienza University of Rome, Via dei Marsi, 78, 00185 Rome, Italy; 2 Department of General Psychology, University of Padova, Via Venezia, 8, 35131 Padova, Italy

**Keywords:** Intolerance of uncertainty, Emotional processing, Adolescence, Longitudinal study, Random Intercept Cross-Lagged Panel Model, Emotion regulation

## Abstract

Previous research on the relation between Intolerance of Uncertainty (IU) and Emotional Processing (EP) during adolescence is limited. The present study aimed to clarify how IU and EP evolve over time, to what extent they are related, and whether changes in one precede or follow changes in the other. A total of 457 Italian adolescents (53.1% girls) aged 11 to 18 years (M = 14.1 ± 2.27) completed the IU and EP scales on three separate occasions three months apart (T1, T2, and T3). Data were modeled using a Random-Intercept Cross-Lagged Panel Model (RI-CLPM) that separates individual differences between individuals from intraindividual changes. Descriptive analyses showed that IU was relatively stable between T1 and T2 and decreased slightly between T2 and T3. Consistent with the view that emotion regulation improves as adolescents develop, EP difficulties were found to decrease between T1 and T2 and between T2 and T3. The RI-CLPM revealed a strong between-person effect, showing that individual differences in IU and EP remained consistent throughout the study. In addition, significant within-person change was found, with adolescents who increased their IU at T1 and T2 also increasing their EP difficulties at T2 and T3, respectively. The opposite effect had a smaller effect size. In sum, our study showed that IU and EP are intertwined in adolescents and that changes in IU precede corresponding changes in EP. These results suggest a priority of change between IU and EP and confirm the relationship between IU and emotion regulation problems in adolescence.

**Supplementary Information:**

The online version contains supplementary material available at 10.1007/s10802-022-01020-1.

## Introduction

Adolescence is a critical transition period characterized by cognitive, physical, psychological, and social changes that make this phase of life highly challenging (Blakemore, 2019; Casey et al., 2010; Powers & Casey, 2015). Adolescents are also at a high risk of developing psychological problems. For example, it has been found that about 13% of children and adolescents worldwide have at least one psychopathology (Polanczyk et al., 2015), the most common of which are anxiety and depressive disorders (de Girolamo et al., 2012; Kessler et al., 2007). Findings from a recent meta-analysis showed that, following the COVID-19 pandemic, prevalence rates of clinically severe anxiety and depression in large juvenile cohorts increased from about 12% to more than 20%, drawing attention to preventing such emotional problems (Racine et al., 2021). Adolescents' emotional problems are also linked to negative life outcomes, such as impairments in personal, family, relational, and academic functioning, and increase the risk of developing and maintaining psychopathologies in adulthood (Copeland et al., 2009). Therefore, it is paramount to gain a deeper understanding of transdiagnostic vulnerability factors in adolescents, such as Intolerance of Uncertainty (IU) and difficulties in Emotional Processing (EP), to create early interventions that specifically address these factors and avoid long-term maladaptive effects (e.g., Bottesi, 2022).

### Intolerance of Uncertainty

IU is the individual's “tendency to be bothered or upset by the (as yet) unknown elements of a situation, whether the possible outcome is negative or not” (Freeston et al., 2020; p. 6) and is characterized by negative beliefs and reactions to uncertainty (e.g., Carleton, 2016). Uncertainty is usually experienced as unpleasant, and people who face it in everyday life tend to enact behaviors aiming to reduce (e.g., excessive information seeking), avoid (e.g., distracting), or remove (e.g., impulsive behaviors) uncertainty distress (Sankar et al., 2017). These strategies are not maladaptive *per se*, but their inflexible use can negatively reinforce uncertainty aversion and desire for predictability (Freeston et al., 2020).

Since emotional and cognitive reactions to uncertainty drive uncertainty-reducing behaviors, this can lead to a vicious cycle that promotes the development and maintenance of IU and ultimately leads to mental health issues (Bottesi et al., 2019a, 2020; Freeston et al., 2020). Indeed, IU is recognized as a transdiagnostic vulnerability factor for psychopathology, including Generalized Anxiety Disorder (GAD), Obsessive-Compulsive Disorder (OCD), social anxiety disorder, panic disorder, health anxiety, depression, Post-Traumatic Stress Disorder (PTSD), eating disorders, substance use disorders, and personality disorders (e.g., Bottesi et al., 2018, 2021; Gentes & Ruscio, 2011; Shihata et al., 2016). Moreover, IU is considered a trans-therapeutic mechanism of change in treatment (McEvoy & Erceg-Hurn, 2015; Norton & Paulus, 2016; Stevens et al., 2018), and evidence about the effectiveness of transdiagnostic psychological treatments targeting IU is growing (Mofrad et al., 2020; Oglesby et al., 2017).

Uncertainty is a core feature of adolescence, as adolescents are confronted with new and unpredictable events and situations (Casey et al., 2010). Moreover, adolescence is characterized by the development of brain areas underpinning cognitive beliefs about the future and uncertain circumstances (i.e., lateral prefrontal cortical areas and anterior cingulate cortex-based networks) (Krain et al., 2006; Steinberg, 2008). Nonetheless, relatively little research has examined IU in adolescents so far. The few studies on this topic highlighted that high IU might pose a serious threat to young people's mental health; indeed, significant relations between IU and psychological problems in adolescents have been found. For example, IU was associated with GAD, social anxiety, separation anxiety, panic disorder, OCD, and depression in non-clinical adolescent groups (Boelen et al., 2010; Laugesen et al., 2003; Read et al., 2013; Wright et al., 2016). More recently, however, increasing attention has been devoted to the relation between IU, externalizing psychopathology, and self-harm, providing further support to the transdiagnostic nature of IU in adolescents (Bottesi et al., 2022). Preliminary evidence also suggests that IU might be a vulnerability factor promoting drinking and alcohol consumption (Oglesby et al., 2017) and restrictive eating behaviors (Konstantellou et al., 2019) in non-clinical and clinical youth samples.

### Emotion Dysregulation and Emotional Processing

Psychopathology is also characterized by poorly regulated emotions (D'Agostino et al., 2016; Sheppes et al., 2015). Emotion regulation abilities begin to develop very early in childhood when infants rely primarily on interactions with caregivers to manage their own emotional states (i.e., co-regulation); subsequently, these abilities are progressively internalized and children become increasingly independent in regulating their emotions (i.e., self-regulation; Bunford, 2020; LoBue et al., 2019). In particular, from childhood to adolescence, some skills that are fundamental to properly monitoring and evaluating one’s emotional states (e.g., executive functions, understanding of emotions, cognitive complexity) increase in maturity, resulting in a wider repertoire of emotion regulation strategies (Zimmermann & Iwanski, 2014).

Adult-like emotion regulation typically develops during adolescence, corresponding with the maturation of the dorsolateral and ventromedial prefrontal cortices (DLPFC and VMPFC, respectively) and their connections with limbic areas (Rawana et al., 2014; Zimmermann & Iwanski, 2014). However, only in late adolescence and emerging adulthood the prefrontal cortex is mature enough to fully support the use of complex, flexible, and more sophisticated emotion regulation strategies (Casey et al., 2010; Powers & Casey, 2015).

The challenges that adolescents typically face make this developmental stage a period of affective turmoil, with a significant increase in emotional reactivity, instability, and a higher frequency and intensity of emotional arousal (Steinberg, 2008). Because the prefrontal cortex has not yet reached full maturity in early adolescence, the ability to adaptively face emotions–especially the negative ones–appears limited, with a high risk of developing emotion dysregulation (Cracco et al., 2017).

Several approaches have been proposed to define emotion dysregulation (Garnefski & Kraaij, 2007; Gratz & Roemer, 2004; Gross & Thompson, 2007; Parker et al., 2001; Rachman, 2001). As a proxy for emotion dysregulation, we focus on EP, a broader term that encompasses emotion dysregulation and other processes also relevant to IU, such as how people experience, categorize, interpret, and deal with emotional experiences (Peluso & Freund, 2019). In the EP framework, emotion regulation is specifically viewed as a naturally occurring habituation process through which emotional experiences are gradually absorbed and reduced to the point where goal-directed activities can proceed without interruption (Rachman, 2001). Successful EP is thought to result in the ability to recognize and describe one's affective states without becoming overly emotionally disturbed; in contrast, unsuccessful EP results in emotion dysregulation in terms of more intense emotional distress states that are permanently activated (e.g., Baker et al., 2012; Greenberg, 2016). Emotional activation interferes with and makes it challenging to engage in goal-directed activities because, in fact, incomplete EP forces the person to function at a high level of arousal (e.g., Baker et al., 2012; Greenberg, 2016).

The most significant mechanisms preventing the spontaneous completion of EP are attempts to control the surge of emotions, such as avoidance of emotional triggers and suppression of emotional experience (e.g., Baker et al., 2012). In this perspective, the inflexible use of behaviors aiming to reduce, avoid, or remove uncertainty (e.g., Sankar et al., 2017) can be viewed as hindering the EP of uncertainty itself, possibly reinforcing uncertainty aversion and desire for predictability. Making dysfunctional appraisals and lacking emotional awareness are the other prominent aspects contributing to emotion dysregulation from an EP standpoint (e.g., Baker et al., 2007).

On the one hand, dysfunctional appraisals (e.g., incorrect evaluations of emotional events) can promote maladaptive behavioral strategies and reinforce distorted beliefs that support the maintenance of psychopathological symptoms in adolescents (e.g., PTSD symptoms; Mitchell et al., 2017); on the other hand, adolescents lacking emotional awareness are deemed to deplete their cognitive resources when trying to make sense of emotional experiences that are ambiguous to them and therefore have fewer resources for developing adaptive emotional responses (e.g., Cracco et al., 2017; Kranzler et al., 2016).

### The Present Study

IU and EP difficulties are transdiagnostic vulnerability factors for psychopathology (Boswell et al., 2013; Ehring & Behar, 2020; Einstein, 2014; Lukas et al., 2018) and potential targets for the treatment of mental health problems in adolescents (e.g., Adams & Gibbons, 2019; Gillett et al., 2018; Kendall et al., 2020; Suveg et al., 2009). This notwithstanding, little research has been conducted to clarify how IU and EP evolve during adolescence, to what extent they are interrelated in adolescents, and whether changes in one precede or follow changes in the other. For example, it has been proposed that IU beliefs may be reinforced by individuals' poor ability to identify emotions, affecting their ability to accurately understand uncertain situations (Abbate-Daga et al., 2011). Other studies suggested that IU beliefs and behaviors (e.g., seeking reassurance or attempts to control uncertainty) may make people prone to have difficulties in EP, which promotes anxiety (Ouellet et al., 2019; Shu et al., 2022). To the best of our knowledge, research on this topic is scarce, based almost exclusively on clinical groups, and hampered by cross-sectional designs that do not allow for determining the temporal link between IU and EP and possibly suggesting a causal relation. Moreover, to date, neither cross-sectional nor longitudinal research has investigated the association between IU and difficulties in EP in adolescent samples.

To complicate things further, research has shown that measures of IU and emotion dysregulation (including EP scales) have trait-like characteristics (i.e., relatively stable over time and across situations). Indeed, IU has been defined as a personality trait (Mahoney & McEvoy, 2012), while the term "EP style" is used to describe that everyone deals with, experiences, and expresses emotional events consistently (e.g., Baker & Berenbaum, 2008; Brintzinger et al., 2021). Given the prominence of trait/style elements, highlighting the dynamics of change and mutual influences between the two constructs is difficult. Luckily, IU and EP are also malleable constructs amenable to change (e.g., through psychotherapy; Baker et al., 2013; McEvoy & Erceg-Hurn, 2015). Thus, observing how IU and EP unfold over time might reveal directional effects, provided that the trait component is controlled for and the analysis focuses on the state-like aspect.

Accordingly, the present study collected data on three occasions (over six months) and used a *Random Intercept Cross-Lagged Panel Model* (RI-CLPM) to disentangle trait and state components. We hypothesized that IU and EP time-invariant trait components would be relatively stable and substantially intercorrelated during the study period. However, we also expected IU and EP states to fluctuate around the participant trait level according to contingent situational demands or intraindividual change. In this regard, the RI-CLPM approach separates within-person change from between-person stability and defines autoregressive and cross-lagged effects between adjacent measurement occasions to highlight reciprocal influences. The autoregressive effects indicate whether a change in IU (or EP) state depends on its past state. Instead, the cross-lagged effects indicate whether a change in the IU state depends on the past change in the EP state and vice versa. Thus, if an IU-to-EP cross-lagged effect is found, then a modification in IU at a specific time point will likely affect the subsequent EP level. Conversely, a significant cross-lagged EP-to-IU effect backs up the hypothesis that a change in EP might affect adolescents’ coping with life uncertainties. Reciprocal relations are also possible if both cross-lagged effects are found.

## Methods

### Participants

The initial sample consisted of 457 Italian (100% Caucasian) adolescents (243 girls, 53.1%) between 11 and 18 years of age (*M* = 14.1 ± 2.27). 45.9% of the participants attended a lower secondary school, and 54.1% upper secondary schools located in a midsized city in northern Italy. Specifically, about the former, 29% attended the first Italian class (6th grade), 39.1% attended the second class (2nd grade), and 31.9% attended the third class (3rd grade). Among high school students, 20.6% attended the first class (9th grade), 21.1% attended the second class (10th grade), 21.1% attended the third class (11th grade), and 37.2% attended the fourth and fifth classes (12th grade). Participants were asked whether they had ever experienced psychological difficulties for which they sought professional help. Among those who responded (*n* = 428), 8.4% reported current or past psychological problems, such as depression, anxiety, neurodevelopmental disorders (e.g., Attention-Deficit/ Hyperactivity Disorder, Specific Learning Disorder), and family or school problems.

### Procedure

The data used in this study were part of a larger research project aiming to investigate the stability of IU and its role as a transdiagnostic vulnerability factor for the onset of psychological problems in adolescence. The study was approved by the Ethics Committee for Psychological Research of the University of Padova and was conducted in accordance with the Declaration of Helsinki. The project was first exposed to school directors before recruiting participants and obtaining their approval. Then, a written informed consent form was signed by 18-year-old participants and, in the case of students younger than 18 years, by their parents or guardians; verbal consent was also obtained from all participants aged 11 to 17.

Adolescents participated in up to three assessment waves (referred to as T1, T2, and T3), during which they filled in an ad hoc online survey including a socio-demographic form and self-report instruments assessing IU, EP, psychopathological symptoms, and different aspects of psychological well-being; however, for the present study, we considered only the questionnaires described below. Data were collected before the outbreak of the COVID-19 pandemic, and a three-month interval between measurements was used. Specifically, the first assessment occurred in November 2018, and the last was in April 2019. Students completed the survey in their school's computer room, and the time taken for each administration was approximately 45/50 minutes. Data collection was anonymous, and we matched the participants' responses from T1 to T3 using an identification code created by each student. The dataset is available as electronic supplementary material.

### Instruments

The *Intolerance of Uncertainty Scale-Revised* (IUS-R; Bottesi et al., 2022; Walker et al., 2010) is a refinement of the IUS-12 with simplified language to be easily read by an average 11-year-old student. It consists of 12 items assessing IU. Respondents are asked to rate how each item applies to themselves on a 5-point Likert scale. Previous research indicated that the Italian version of the IUS-R has an excellent internal consistency (Cronbach’s α = 0.90), a good one-month test–retest reliability (rtt = 0.74), and the total score was significantly correlated with anxiety, depression, and behavioral and emotional problems (Bottesi et al., 2019b, 2022). In the present study, Cronbach’s α coefficients for the total score were 0.84, 0.89, and 0.90 at T1, T2, and T3, respectively.

The *Emotional Processing Scale* (EPS; Baker et al., 2015) is a 25-item questionnaire to assess EP difficulties in clinical and non-clinical populations. Each item uses a 10-point visual analog rating scale, from 0 (*completely disagree*) to 9 (*completely agree*). The EPS has been the subject of numerous psychometric investigations. The results generally indicate the overall scale has excellent internal consistency: 0.92 in the original version (Baker et al., 2015), 0.91 in the Iranian, Spanish, and French versions (Gay et al., 2019; Kharamin et al., 2021; Orbegozo et al., 2017), and 0.94 in the Italian version (Lauriola et al., 2021). In the present study, Cronbach’s α coefficients for the total score were 0.92, 0.92, and 0.93 at T1, T2, and T3, respectively.

*Significant Life Events*: considering the longitudinal study design, at the second (T2) and third (T3) administrations participants were asked whether notable life events (both positive and negative) had occurred over the past three months and, if so, what such events were. Among those who answered (*n*_T2_ = 268 and *n*_T3_ = 263), most participants did not report any significant life event (72.4% at T2 and 65% at T3). Events were then classified as positive (+1), negative (-1), or neutral/unclear (0) by two independent raters. According to Landis and Koch standards (1977), Cohen's κ inter-rater reliability indicated nearly perfect agreement for T1-T2 and T2-T3 events (κ_T1-T2_ = 0.94; κ_T2-T3_ = 0.88). Events were used as covariates in a sensitivity analysis to assess the robustness of our findings to possible confounding factors acting at the intra-individual level. To this end, disagreements in event coding were resolved through discussion, and the dataset was collaboratively modified to reach a complete agreement.

### Statistical Analysis

#### Preliminary Analyses

Total scores at each time point of the study were calculated for the IUS-R and EPS. The data were checked for univariate normality using the Shapiro-Wilk's test. Given this test’s high false positive rate with relatively large samples, we also evaluated the acceptability ranges of skewness and kurtosis according to the commonly accepted rule of thumbs (i.e., between ± 0.5 and ± 3, respectively). Because attrition can affect the interpretation of study results, a missing value analysis was carried out. Little’s MCAR test was performed to rule out that missing data patterns violated the assumptions of non-random distribution. Under this assumption, model parameters can be safely estimated using all available cases, with missing values imputed using Full Information Maximum Likelihood (FIML). The averages obtained in the IU and EP scores at T1, T2 and T3 were compared by t-test. Beyond significance, we evaluated the effect size using Cohen's *d*, with small effect sizes corresponding to *d* values around 0.2, medium effect sizes corresponding to *d *values around 0.5, and large effect sizes corresponding to *d *values around 0.8 (Cohen, 1988). Standardized indirect effects in the main analyses can also be interpreted according to Cohen’s standards.

#### Main Analyses

RI-CLPM (Mulder & Hamaker, 2021) was employed to separate within-person from between-person IU and EP components. The analyses were conducted using the *lavaan* R package (Rosseel, 2012). Because we measured the two constructs on three occasions, six latent variables (i.e., IU_W1_, IU_W2_, IU_W3,_ EP_W1_, EP_W2_, EP_W3_) were formed to shape the within-person component. Two random intercept factors (i.e., IU_B_ and EP_B_) were created to capture between-person variance. Each wave's IU and EP scores were set to load the corresponding random intercept factor with a loading fixed to one. Within-person variability represents the putative dynamic, state-like component and is measured by the size of autoregressive and cross-lagged path coefficients connecting adjacent within-subject factors. Between-person variability represents an enduring, trait-like process, and the covariance between the random intercepts determines its size. For interpretation, the autoregressive paths in RI-CLPM indicate the stability of IU and EP states, not accounted for by the corresponding trait, a sort of "inertia" or carryover effect due to the consistency of situational demands (e.g., no uncertainty or emotionally salient episodes) or mere temporal proximity of adjacent IU and EP states. In contrast, the cross-lagged paths indicate how the participants’ deviation from their trait IU level in each wave was predicted by variations from their trait EP on the previous wave after controlling for the carryover effects, and vice versa. RI-CLPM is an extension of the more basic *Cross-Lagged Panel Model* (CLPM), in which within- and between-person levels are entangled. So, the estimated cross-lagged coefficients in CLPM represent a mixture of state-like and trait-like processes. If the trait-like variance is the only source involved in the reciprocal influences between IU and EP, the CLPM model is expected to fit the data equally well as the RI-CLPM. Conversely, a poorer fit of the CLPM model relative to the RI-CLPM indicates that isolating the trait-like component of IU and EP is vital to representing how the state-like aspects of the two constructs are interrelated. Because the CLPM is nested in the RI-CLPM, the fit of the two models can be compared using the chi-square difference test. If the test is not significant, the more parsimonious CPLM model is preferred. Conversely, if the test turns out significant, RI-CLPM is retained.

## Results

### Descriptive Analyses

Table 1 shows the study variables' means, standard deviations, and correlations. The Shapiro-Wilk's test was significant for IUS-R and EPS scores at all time points (all *p*-s < 0.001). However, the skewness and kurtosis indicated that the data were quite symmetrical, and the extreme values were similar to those expected for a normal distribution. Missing data were observed, ranging from a low of 9 for the IUS-R at T1 to a high of 66 for the EPS at T3. Little’s MCAR test was not significant at *p* < 0.01 (χ^2^ = 81.66, df = 81, *p* = 0.459), suggesting that missingness was completely random. This finding suggests that the risk of attrition bias was negligible. Analog to test–retest coefficients, the correlations across times among IUS-R scores and those among EPS scores were the highest reported in Table 1. EPS and IUS-R scores were positively intercorrelated within each time point (*r* = 0.42, *r* = 0.51, and *r* = 0.51 at T1, T2, and T3 respectively). The lowest correlations were between different scales administered at different time points.

**Table 1:** Descriptive statistics and correlation coefficients between IU and EP

	Correlation Coefficients					
1. IUS-R (T1)	–					
2. IUS-R (T2)	0.62	–				
3. IUS-R (T3)	0.50	0.70	–			
4. EPS (T1)	0.42	0.37	0.31	–		
5. EPS (T2)	0.41	0.51	0.45	0.63	–	
6. EPS (T3)	0.34	0.42	0.51	0.55	0.68	–
	1.	2.	3.	4.	5.	6.

	Descriptive Statistics					
*N*	448	429	407	431	407	391
Mean	2.66	2.66	2.59	3.52	3.25	3.15
*SD*	0.74	0.82	0.86	1.71	1.82	1.76
Skewness	0.23	0.14	0.16	0.14	0.06	0.13
Kurtosis	-0.15	-0.30	-0.18	-0.76	-0.74	-0.31

All correlations were statistically significant at *p* < 0.001

*IUS-R* Intolerance of Uncertainty Scale-Revised, *EPS* Emotional Processing Scale, *T1* First measurement occasion, *T2* Second measurement occasion, *T3* Third measurement occasion

Table 2 shows the results of regression analyses in which age and sex were used as predictors of IUS-R and EPS scores. Older age was associated with lower IU, but only at baseline. Regarding EP difficulties, older age was associated with higher EPS scores, but the conventional levels of statistical significance were reached only at T3. Girls obtained systematically higher scores than boys in the EPS scores on all measurement occasions. Collectively, age and sex explained a very small proportion of the variance in the EPS scores (up to a maximum of 3%).

**Table 2:** Age and sex differences in IU and EP at different measurement occasions (T1, T2, and T3)

Predictor	*B*	β	*t*	*p*	*B*	β	*t*	*p*
	IUS-R (T1)				EPS (T1)			
Age	-0.04	-0.11	-2.42	0.016	0.06	0.08	1.65	0.099
Sex (Girls-Boys)	0.10	0.14	1.49	0.136	0.45	0.26	2.72	0.007
	*R*^2^ = 0.02				*R*^2^ = 0.02			
	IUS-R (T2)				EPS (T2)			
Age	0.00	0.01	0.16	0.870	0.08	0.10	1.95	0.052
Sex (Girls-Boys)	0.05	0.06	0.60	0.547	0.47	0.26	2.60	0.010
	*R*^2^ = 0.00				*R*^2^ = 0.03			
	IUS-R (T3)				EPS (T3)			
Age	0.00	0.01	0.21	0.830	0.13	0.16	3.17	0.002
Sex (Girls-Boys)	0.00	0.00	0.00	0.997	0.10	0.06	0.58	0.565
	*R*^2^ = 0.00				*R*^2^ = 0.03			

*IUS-R* Intolerance of Uncertainty Scale-Revised, *EPS* Emotional Processing Scale, *T1* First measurement occasion, *T2* Second measurement occasion, *T3* Third measurement occasion

Paired sample t-tests (Table 3) showed that the overall sample IU level was stable between T1 and T2 to decrease slightly between T2 and T3. Conversely, EP difficulties decreased between T1 and T2 and between T2 and T3. Although the effect sizes were at best small, indicating a general scenario of high stability over the study period, the longitudinal trend is consistent with the view that emotion regulation abilities tend to improve with adolescent development.

**Table 3:** Tests of significant differences between adjacent measurement occasions for IU and EP

Comparison	Mean difference	Student's *t*	df	*p*	Cohen’s *d*
IUS-R (T1–T2)	0.04	0.11	425	0.909	0.01
IUS-R (T2–T3)	0.07	2.15	396	0.032	0.11
IUS-R (T1–T3)	0.08	1.90	402	0.058	0.09
EPS (T1–T2)	0.28	3.70	401	< 0.001	0.18
EPS (T2–T3)	0.14	1.83	370	0.068	0.09
EPS (T1–T3)	0.41	4.92	386	< 0.001	0.25

*IUS-R* Intolerance of Uncertainty Scale-Revised, *EPS* Emotional Processing Scale, *T1* First measurement occasion, *T2* Second measurement occasion, *T3* Third measurement occasion

Last we examined whether life events affected IU and EP change between adjacent measurement occasions. As seen in Table 4, life events occurring between T1 and T2 did not impact IUS-R or EPS T2-T1 change scores. Life events occurring between T2 and T3 slightly increased adolescents’ IUS-R scores (*p* = 0.070). Again, the effect sizes were null-small, indicating life events reported by adolescents had a negligible effect on IU and EP changes across time.

**Table 4:** Significant life events reported by adolescents between T1 and T2, and between T2 and T3, predict subsequent changes in IU and EP scores between adjacent measurement occasions

Predictor	*B*	β	*t*	*p*	*B*	β	*t*	*p*
	IUS-R (T2-T1 change)				EPS (T2-T1 change)			
Life Events (T1 → T2)	0.02	0.01	0.18	0.860	-0.07	-0.02	-0.35	0.730
	*R*^2^ = 0.00				*R*^2^ = 0.00			
	IUS-R (T3-T2 change)				EPS (T3-T2 change)			
Life Events (T1 → T2)	-0.12	-0.07	-1.36	0.175	0.06	0.02	0.29	0.769
Life Events (T2 → T3)	0.14	0.10	1.85	0.065	-0.02	-0.01	-0.10	0.923
	*R*^2^ = 0.01				*R*^2^ = 0.00			

*IUS-R* Intolerance of Uncertainty Scale-Revised, *EPS* Emotional Processing Scale, *T1* First measurement occasion, *T2* Second measurement occasion, *T3* Third measurement occasion

### Standard Cross-Lagged Panel Model (CLPM)

**Fig. 1:**
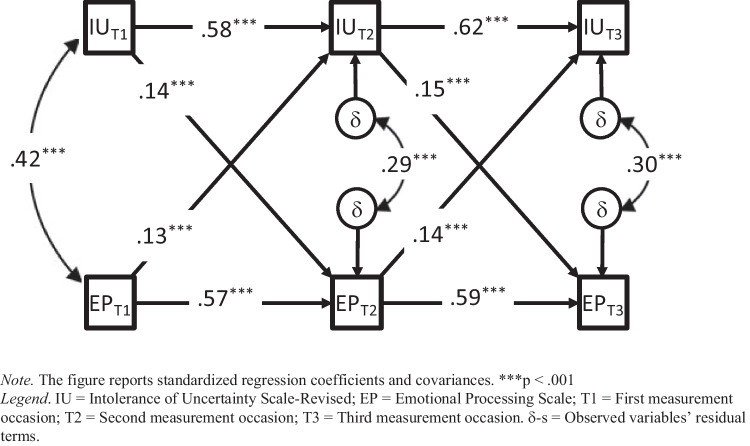
CLPM of IU and EP

A standard CLPM was calculated to assess the “gross” bidirectional relations between IU and EP. Except for RMSEA, all fit indexes were acceptable to good (χ^2^ = 26.95, df = 4, *p* < 0.001, CFI = 0.980, TLI = 0.923, RMSEA = 0.112, SRMR = 0.027). Because the time lags between waves were approximately the same, we restricted the autoregressive, the cross-lagged paths, and the within-time correlations between IU and EP to be time-invariant. The constrained CLPM showed acceptable-to-good fit (χ^2^ = 46.98, df = 10, *p* < 0.001, CFI = 0.967, TLI = 0.950, RMSEA = 0.090, SRMR = 0.075). However, the chi-square difference test indicated a significant loss of fit (Δχ^2^ = 20.029; df = 6; *p* = 0.003). The inspection of the model modification indices suggested removing the equality constraint for the within-time correlation between IU and EP at T1. This modification improved the fit of the constrained model (χ^2^ = 29.69, df = 9, *p* < 0.001, CFI = 0.982, TLI = 0.969, RMSEA = 0.071, SRMR = 0.029) and made it not significantly different from the unconstrained one (Δχ^2^ = 2.74; df = 5; *p* = 0.740). The analysis supported the longitudinal invariance of the autoregressive and cross-lagged paths, and the partial invariance of within-time correlations at T2 and T3. Standardized parameter estimates are reported in Fig. 1.

Significant positive cross-lagged effects emerged either from IU at T1 and T2, to EP at T2 and T3, or from EP at T1 and T2, to IU at T2 and T3, suggesting reciprocal influences over time. As a result, adolescents who reported higher IU levels (or difficulties in EP) at T1 and T2 relative to their peers tended to also express more difficulties in EP (or report higher IU levels) at T2 and T3. To determine whether the cross-lagged effects from IU to EP differed in magnitude from those from EP to IU, we constrained the corresponding cross-lagged paths to be equal. Model fit still was acceptable (χ^2^ = 43.65, df = 10, *p* < 0.001, CFI = 0.970, TLI = 0.955, RMSEA = 0.086, SRMR = 0.053), but the cross-lagged parameters could not be assumed to be equal (Δχ^2^ = 13.96, df = 1, *p* < 0.001). Despite similarities in standardized estimates (Fig. 1), the statistical effect from IU to EP was greater than that from EP to IU.

### Random Intercept Cross-Lagged Panel Model (RI-CLPM)

The CLPM model conflated within- and between-person variance and the parameters reported in Fig. 1 reflected how well the participants’ rank in the EP and IU sample distributions at T2 and T3 could be predicted based on prior knowledge of their rank at T1 and T2, respectively. In contrast, RI-CLPM parameters show how predictable within-person fluctuations are with respect to one's own scores. The model fit of the unconstrained RI-CLPM was near perfect (χ^2^ = 0.069, df = 1, *p* = 0.792, CFI = 1.000, TLI = 1.007, RMSEA = 0.000, SRMR = 0.002). As in previous analyses, we restricted the autoregressive, the cross-lagged paths, and the within-time correlations between IU and EP to be time-invariant. The constrained RI-CLPM (χ^2^ = 3.021, df = 7, *p* = 0.883, CFI = 1.000, TLI = 1.008, RMSEA = 0.000, SRMR = 0.013) fitted the data as equally well as the unconstrained model (Δχ^2^ = 2.95, df = 6, *p* = 0.815). Because the CLPM is nested within the RI-CLPM (i.e., the CLPM can be derived from the RI-CLPM constraining the random-intercept variance to 0), we also compared the two approaches to determine whether accounting for trait-level effects improved model fit. Indeed, the RI-CLPM model was a significant improvement in fit compared to CPLM (Δχ^2^ = 26.66, df = 2, *p* < 0.001).

**Fig. 2:**
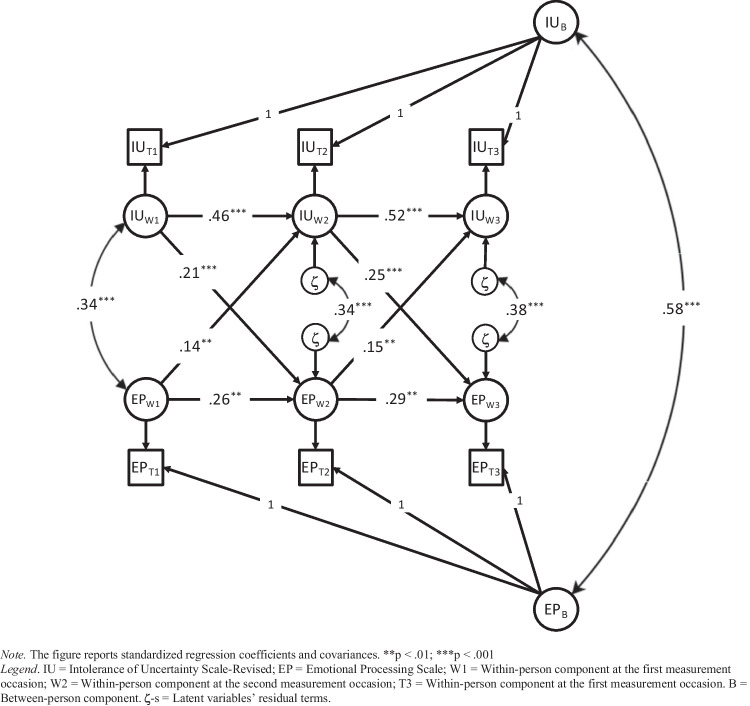
RI-CLPM of IU and EP

Figure 2 shows the standardized RI-CLPM parameters. The between-person correlation between IU and EP was large and positive, indicating that adolescents high on trait IU across measurement occasions chronically experienced more difficulties in EP than adolescents low on IU. At the within-person level, significant positive concurrent associations were also found between IU and EP. Thus, adolescents who scored higher (or lower) than their expected trait IU level also tended to score higher (or lower) than their expected EP level on T1, T2, and T3. Positive within-person cross-lagged coefficients connected IU to EP, indicating that adolescents' deviations from their habitual EP scores were predicted by corresponding shifts in IU at the previous time point. In other words, adolescents who scored higher than they typically would on IU were more likely to experience higher EP difficulties than they typically would at the next wave. Conversely, the within-person cross-lagged coefficients from EP to IU were weaker than those previously described and marginally significant or not at all significant. This finding indicated that adolescents' deviations from their habitual IU level were less strongly predicted by corresponding shifts in EP at the previous wave. The analysis also yielded significant positive carry-over stability coefficients of IU and EP state components between waves.

As in previous analyses, we formally tested whether the cross-lagged effects from IU to EP differed from those from EP to IU. Accordingly, we constrained the corresponding cross-lagged paths to be equal. Although the model fit still was acceptable (χ^2^ = 43.65, df = 10, *p* < 0.001, CFI = 0.970, TLI = 0.955, RMSEA = 0.086, SRMR = 0.053), the cross-lagged parameters from IU to EP could not be assumed to be equal to those from EP to IU (Δχ^2^ = 10.55, df = 1, *p* < 0.01). Hence, the analysis supported the view that intra-individual changes in EP difficulties were secondary to changes in IU.

### Sensitivity Analyses

Because our sample included some adolescents who reported psychological problems for which they sought professional help, potential mental health disorders might have had a high likelihood of being associated with IU and EP, inflating their associations. To assess the impact of self-reported psychological problems on our results, we retested the RI-CLPM model with 392 participants who did not report any problem. As in previous analyses, the model fit of the unrestricted RI-CLPM was nearly perfect (χ^2^ = 0.338, df = 1, *p* = 0.561, CFI = 1.000, TLI = 1.011, RMSEA = 0.000, SRMR = 0.005). When we constrained the autoregressive, the cross-lagged paths, and the within-time correlations between IU and EP to be time-invariant, the constrained model (χ^2^ = 4.319, df = 7, p = 0.742, CFI = 1.000, TLI = 1.007, RMSEA = 0.000, SRMR = 0.017) fitted the data as the unconstrained model (Δχ^2^ = 3.98, df = 6, *p* = 0.679). The model’s coefficients were very similar to those estimated in the main analysis. Important for the main purpose of the study, the cross-lagged coefficients were only negligibly smaller than in the main analysis (i.e., 0.19, 0.23, 0.14, and 0.15 Vs. 0.21, 0.25, 0.14, and 0.15, respectively). Consequently, the equality of cross-lagged paths from IU to EP versus EP to IU was rejected again (Δχ^2^ = 7.00, df = 1, *p* < 0.01), supporting the view that intra-individual changes in EP difficulties were secondary to corresponding changes in IU in a sample of putatively healthy adolescents.

**Fig. 3:**
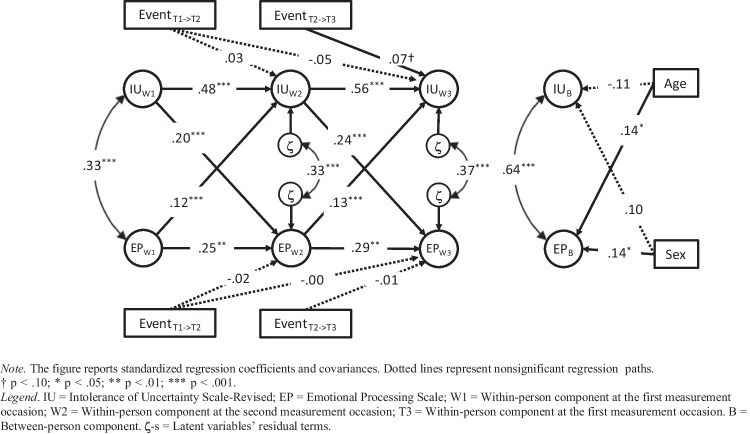
Schematic representation of the RI-CLPM of IU and EP, with events as covariates of within-person factors and age and sex as covariates of the between-person factors

Before concluding, we addressed the question of whether life events occurring during the study might have impacted our results. We modified the RI-CLPM adding the life events occurring between T1 and T2 and T2 and T3 as covariates of the within-person factors and age and sex as covariates of the between-person factors (Fig. 3). Model’s fit was excellent (χ^2^ = 27.03, df = 21, *p* = 0.170, CFI = 0.995, TLI = 0.990, RMSEA = 0.025, SRMR = 0.024). The events that occurred during the study and were reported as significant by adolescents did not predict the subsequent within-person EP factors. We found a marginally significant association between events occurring between T1 and T2 and within-person factor IU at T3. Age and sex did not predict the IU between-person factor, but were associated with EP. Despite the inclusion of the covariates, the autoregressive and cross-lagged coefficients replicated those obtained from the model without the covariates (Compare Figs. 2 and 3).

## Discussion

The current study examined the temporal associations between IU and EP difficulties at the intra-individual level while adjusting for inter-individual variability. To our knowledge, no previous research has specifically investigated the temporal link between these two constructs in adolescence through a longitudinal design, despite both IU and EP difficulties being well-documented transdiagnostic vulnerability factors to psychopathology (e.g., D’Agostino et al., 2016; Sheppes et al., 2015; Shihata et al., 2016).

Initially, the results obtained from a standard CLPM analysis revealed a reciprocal association between IU and EP over 6 months. Indeed, the cross-lagged effects from IU to EP were only marginally different from those linking EP to IU. These findings suggested that adolescents reporting higher IU levels than their peers also experienced more EP difficulties during the study period. Conversely, those experiencing more EP difficulties than their peers reported higher IU levels. Highlighting reciprocal influences between IU and EP difficulties expands on previous research, in which various characteristics underpinning emotion dysregulation were found to be correlated with maladaptive uncertainty beliefs and behaviors (Abbate-Daga et al., 2011; Ouellet et al., 2019; Shu et al., 2022). Nevertheless, these studies used cross-sectional designs, and therefore the direction of the associations remained unexplained. It is worth noting, however, that the literature has repeatedly defined IU and EP as relatively stable traits or cognitive styles (e.g., Baker & Berenbaum, 2008; Brintzinger et al., 2021; Mahoney & McEvoy, 2012). Accordingly, reciprocal relations were not entirely unexpected even in a longitudinal study, especially one of limited duration like ours.

The primary goal of our study was to investigate the temporal dynamics of the relation between IU and EP during adolescence, precisely whether a change in one can anticipate a change in the other. As pointed out elsewhere (Mulder & Hamaker, 2021), a CLPM analysis conflates between-person and within-person effects, with the latter representing “true” intra-individual change. Thus, we reanalyzed our data according to a RI-CLPM approach. The new analyses revealed that, when longitudinal associations were tested at the intra-individual level, a change in IU predicted subsequent changes in EP to a greater extent than a change in EP predicted intra-individual change in IU. This is the study’s main result, a conclusion consistent with the notion that uncertainty aversion, negative beliefs about uncertainty, and uncertainty-reducing behaviors can hinder the EP of uncertainty itself (Bottesi et al., 2020; Freeston et al., 2020), thus contributing to making adolescents more prone to experience uncertainty distress – i.e., ‘the subjective negative emotions experienced in response to the as yet unknown aspects of a given situation’ (Freeston et al., 2020, p. 6). These emotions may include anxiety, frustration, anger, sadness, etc., which may further explain why difficulties dealing with uncertainty may lead to difficulties processing emotions (Ouellet et al., 2019; Shu et al., 2022).

RI-CLPM and CLPM led to somewhat different conclusions regarding the size and the interpretation of the cross-lagged effects linking IU with EP. To discern between seemingly contrasting findings, it is worth noting that the reciprocal influences that we observed in CLPM analyses were inflated by the between-person variance, which refers to how changes in the group mean of one variable (e.g., IU) are associated with corresponding changes in the group mean of the other variable (e.g., EP). Usually, this trend is interpreted in terms of stable personal characteristics (Curran & Bauer, 2011), such as trait IU and EP style in the context of the present study. Therefore, the positive cross-lagged effects in the CLPM analyses merely indicated that an increase (or decrease) in the average IU (or EP) produced a corresponding average level change in EP (or IU). For example, our descriptive analysis showed that both IU and EP difficulties tended to decrease steadily from T1 to T3, reflecting the average sample improvement in both constructs during the study period. This result is in line with literature suggesting that uncertainty tolerance and emotion regulation are subject to improvements during adolescence due to the maturation of the brain areas underpinning such processes (e.g., Powers & Casey, 2015; Read et al., 2013).

The present study is not free from limitations. First, the time lag we chose was arbitrary: we covered six months to obtain interpretable results over an entire school year. However, the relations between IU and EP may change using larger time intervals. Thus, replicating these findings through the conduction of more extended longitudinal studies, possibly tracking variations during transition periods (e.g., beginning and end of secondary school; see Dugas et al., 2012), is highly recommended. Second, despite the longitudinal design and the insights gained on the direction of effects, we acknowledge that no causal conclusion can be drawn, given that it is impossible to exclude that some of the observed associations may be due to variables that were not measured in the current study (e.g., neuroticism, distress tolerance, or the “p factor” of psychopathology). For example, neuroticism (i.e., the tendency to experience and over-react to negative emotions) may account for the complex interplay between the constructs under investigation, since both IU and difficulties regulating emotions are considered as predispositions originating from this personality dimension (Carleton, 2016; Silverman et al., 2019). Therefore, future research is needed to verify our findings in clinical samples of adolescents with high severity of persistent negative emotions. If replicated, our results may suggest that IU should be prioritized in preventive interventions and treatments targeting adolescents with mental disorders. Third, the data were based on self-reported scales; thus, shared method and informant variance might have inflated the identified effects. Nonetheless, the cross-lagged approach somewhat reduced this concern by accounting for autoregressive effects. Although our findings were generally consistent with prior studies, future research will benefit from using multiple methods designs (e.g., observational) and multi-informant measures. Finally, we did not collect data about the socioeconomic status of participants. Future studies should also consider this variable since it appears to predict mental health problems in children and adolescents (e.g., Reiss, 2013).

Bearing the shortcomings mentioned above in mind, findings from the present study may tentatively provide helpful theoretical and clinical hints. IU and EP are malleable constructs (Baker et al., 2013; McEvoy & Erceg-Hurn, 2015); therefore, contingent situations (or maturation in a developmental perspective) can shift an adolescent’s level from their usual reference point at a given time. This within-person change is usually interpreted as state-like characteristics (Curran & Bauer, 2011). In this perspective, the cross-lagged effects linking IU to EP in the RI-CLPM analyses turned out more robust than those linking EP to IU, indicating that a positive change in IU predicted a subsequent positive change in difficulties processing emotions, while the opposite interpretation of within-person effects, although possible and even found in the data, was of minor relevance.

Notably, the RI-CLPM performed better than the CLPM in model fit, emphasizing the need to go beyond trait-level changes in IU and EP to better understand their mutual relation during adolescence. Future research replicating these findings is warranted. Indeed, gaining further knowledge about the co-occurrence between IU and difficulties in EP would allow setting the frame for larger studies aiming to explore their joint role in the development of psychopathology (anxiety and depression in particular) during adolescence. According to the "maladaptive shift model" (Zimmermann & Iwanski, 2014), emotion dysregulation is characterized by a dysfunctional shift in adolescence, and chronic difficulty in regulating emotions may have long-term consequences for adolescents' mental health (e.g., Compas et al., 2017; Klein et al., 2022; McLaughlin et al., 2011). Consequently, some scholars maintain that emotion dysregulation may partly explain the peak of psychopathology during adolescence (Cracco et al., 2017; Mendle, 2014). Following these considerations and in light of present findings, recognizing a priority of change between IU and EP difficulties would be advantageous for identifying prospective targets for prevention and treatment interventions. Indeed, if EP difficulties arise because of behaviors aiming to avoid or suppress the emotional experience of uncertainty, then addressing uncertainty aversion and dysfunctional beliefs about IU could facilitate EP, thereby promoting better emotion regulation (Ouellet et al., 2019). If EP difficulties may, in turn, prevent adolescents from coping with uncertainty distress, it is essential to design prevention programs aiming to encourage them to expose themselves to uncertainty safely. For example, Mofrad et al. (2020) claimed that attuning one’s uncertainty by evoking objectively ‘safe’ uncertainty (e.g., by changing a routine) may benefit people high in IU. Moreover, they suggested that providing informal and playful ways to experience uncertainty (e.g., tasting unusually flavored or unlabeled foods) and beginning to label and understand the bodily sensations associated with uncertainty may represent a viable way to promote a positive attitude towards uncertainty, thus helping people tolerating, embracing, and accepting it (Mofrad et al., 2020).

Such an approach may hold great promise from a prevention perspective to adopt in adolescence, considering that IU may be a phase-specific feature of adolescence itself (Bottesi et al., 2022) and that relations with uncertainty pre-verbally develop in infancy when perceiving uncertainty as ‘unsafe’ is advantageous from an evolutionary standpoint (Brosschot et al., 2018). Building experience about uncertainty as a non-threatening feeling may contrast the development of uncertainty distress and dysfunctional IU beliefs and promote adaptive EP, thus ultimately preventing the onset of psychopathology, anxiety, and depression in adolescents.

## Supplementary Information

Below is the link to the electronic supplementary material.

Supplementary file1 (SAV 80 KB)

## Data Availability

The dataset is available as electronic supplementary material.
